# Integrating machine learning and artificial intelligence in life-course epidemiology: pathways to innovative public health solutions

**DOI:** 10.1186/s12916-024-03566-x

**Published:** 2024-09-02

**Authors:** Shanquan Chen, Jiazhou Yu, Sarah Chamouni, Yuqi Wang, Yunfei Li

**Affiliations:** 1 Faculty of Epidemiology and Population Health, London School of Hygiene & Tropical Medicine, Keppel Street, London, WC1E 7HT UK; 2 Department of Medicine and Therapeutics, The Chinese University of Hong Kong, Hong Kong SAR, China; 3 Department of Computer Science, University College London, London, WC1E 6BT UK; 4 Department of Neurobiology, Care Sciences and Society, Karolinska Institutet, Stockholm, 171 64 Sweden

**Keywords:** Machine learning, Artificial intelligence, Life course, Epidemiology, Public health

## Abstract

The integration of machine learning (ML) and artificial intelligence (AI) techniques in life-course epidemiology offers remarkable opportunities to advance our understanding of the complex interplay between biological, social, and environmental factors that shape health trajectories across the lifespan. This perspective summarizes the current applications, discusses future potential and challenges, and provides recommendations for harnessing ML and AI technologies to develop innovative public health solutions. ML and AI have been increasingly applied in epidemiological studies, demonstrating their ability to handle large, complex datasets, identify intricate patterns and associations, integrate multiple and multimodal data types, improve predictive accuracy, and enhance causal inference methods. In life-course epidemiology, these techniques can help identify sensitive periods and critical windows for intervention, model complex interactions between risk factors, predict individual and population-level disease risk trajectories, and strengthen causal inference in observational studies. By leveraging the five principles of life-course research proposed by Elder and Shanahan—lifespan development, agency, time and place, timing, and linked lives—we discuss a framework for applying ML and AI to uncover novel insights and inform targeted interventions. However, the successful integration of these technologies faces challenges related to data quality, model interpretability, bias, privacy, and equity. To fully realize the potential of ML and AI in life-course epidemiology, fostering interdisciplinary collaborations, developing standardized guidelines, advocating for their integration in public health decision-making, prioritizing fairness, and investing in training and capacity building are essential. By responsibly harnessing the power of ML and AI, we can take significant steps towards creating healthier and more equitable futures across the life course.

## Background

Life-course epidemiology is a field of study that examines the long-term effects of biological, behavioral, and social exposures during gestation, childhood, adolescence, and adulthood on the development of chronic diseases later in life [1]. This approach recognizes that health and disease are influenced by the complex interplay of various factors across an individual’s life span and that the timing and duration of these exposures can have critical implications for future health outcomes [1].

**Fig. 1:**
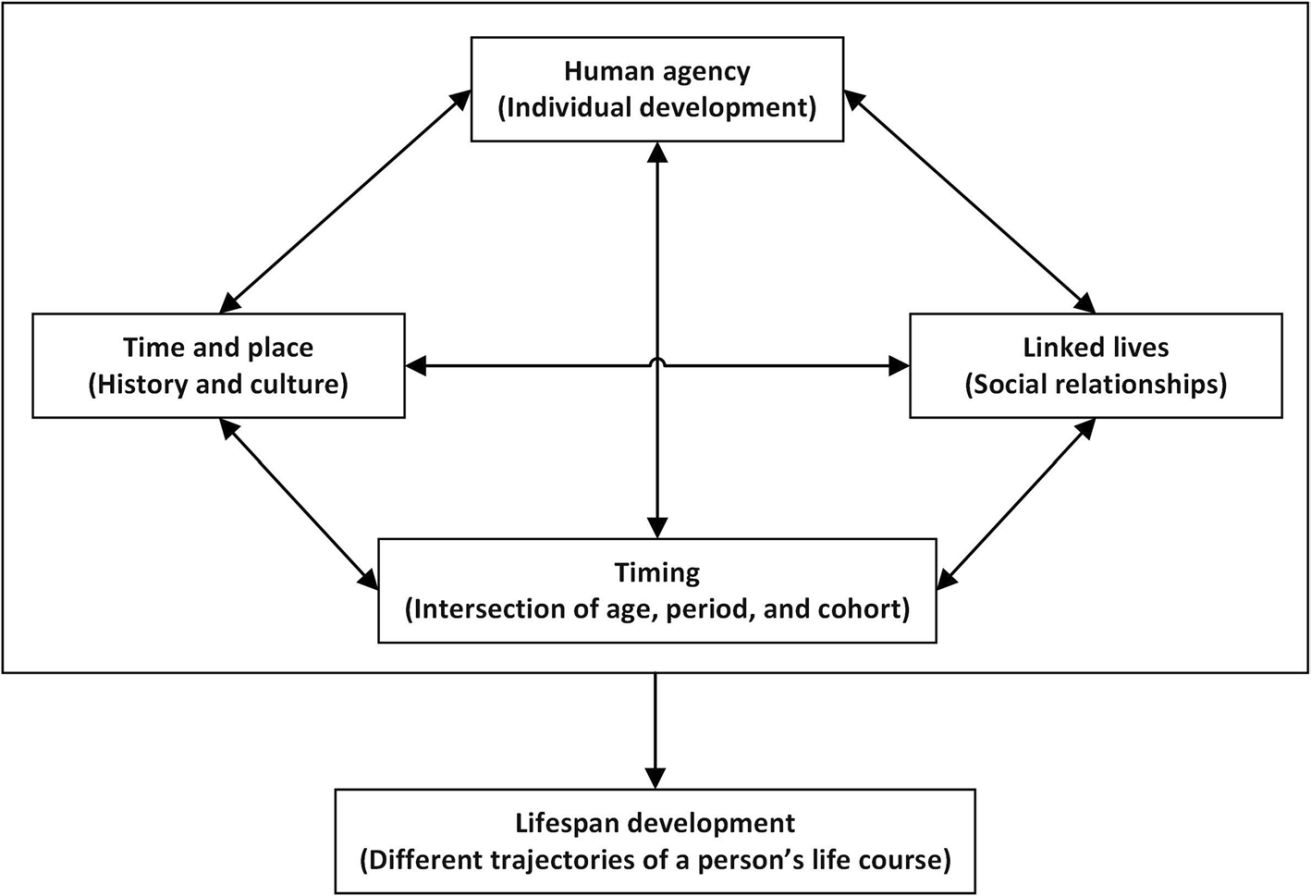
Five principles of the life-course approach proposed by Elder and Shanahan

The importance of life-course epidemiology in understanding chronic diseases lies in its ability to provide a comprehensive framework for investigating the origins and trajectories of these conditions. As defined by Elder and Shanahan, the life-course approach is based on five key principles: lifespan development, agency, time and place, timing, and linked lives (Fig. 1) [2]. Lifespan development recognizes that human development and aging are ongoing processes that occur throughout an individual’s life, rather than being limited to specific stages. Agency acknowledges that individuals have the capacity to make choices and take actions that shape their lives, albeit within the constraints of their environmental, social, and historical contexts. The principle of time and place emphasizes that each person’s life course is embedded within and influenced by the specific historical era and location in which they live. Timing is crucial, as the same events and behaviors can have varying effects depending on when they occur in an individual’s life course. Finally, linked lives underscores the interconnectedness of human experiences, as people influence each other through shared and interdependent relationships. By applying these principles, researchers can identify sensitive periods and critical windows during which interventions may be most effective in preventing or mitigating the risk of chronic diseases.

In recent years, machine learning (ML) and artificial intelligence (AI) have emerged as powerful tools in epidemiological research. ML is a subfield of AI which refers to the ability of computers to draw conclusions (ie, learn) from data without being directly programmed and builds from traditional statistical methods [3]. These techniques offer the ability to handle vast amounts of complex, high-dimensional data, identify intricate patterns and associations, and develop predictive models that can inform personalized interventions and public health strategies. ML and AI can integrate multiple data types, such as electronic health records (EHRs), genomic data, and environmental exposures, to provide a more comprehensive understanding of the factors contributing to health outcomes across the life course. Moreover, advanced ML and AI techniques can analyze multimodal data, including structured and unstructured text, audio, image, and video data, enabling the examination of diverse information sources such as MRI scans, X-rays, recordings of heartbeats or respiratory sounds, and physical activity and behavioral patterns. Furthermore, these approaches can enhance causal inference methods, allowing researchers to better estimate the effects of exposures on health outcomes in observational settings.

The integration of ML and AI techniques in life-course epidemiology has the potential to revolutionize our understanding of the complex determinants of diseases and inform the development of more targeted and effective public health interventions. By leveraging the power of these innovative tools, researchers can uncover novel risk factors, identify critical windows for intervention, and predict individual disease trajectories with greater precision.

This perspective aims to summarize the current applications and discuss future potential and challenges of integrating ML and AI in life-course epidemiology research, and to discuss the applications of such technologies to advance public health solutions. This perspective will also discuss the benefits and limitations of current applications, highlight opportunities for identifying sensitive periods, modeling complex interactions, predicting disease risk trajectories, and enhancing causal inference methods. Further, it will address the challenges and ethical considerations associated with the use of ML and AI in life-course research, and provide recommendations for future directions.

## Current applications of ML and AI in epidemiology

ML and AI have been increasingly applied in various areas of epidemiological studies, demonstrating their potential to advance our understanding of health and diseases. These techniques offer several key benefits, including the ability to handle large, complex datasets, identify intricate patterns and associations, and develop accurate predictive models.

One notable application of ML and AI in epidemiology is in the prediction of cardiovascular disease risk. Researchers have developed ML models that integrate clinical, genetic, and lifestyle factors to predict an individual’s risk of developing cardiovascular disease [4–7]. For example, a study utilized 216,152 retinal photographs from datasets in South Korea, Singapore, and the United Kingdom to train and validate deep learning algorithms [6]. The retinal photograph-derived coronary artery calcium scores were found to be comparable to those measured by CT scans [6]. Ward et al. (2020) used EHRs of 262,923 individuals to train and validate ML models, demonstrating performance that was comparable to or better than traditional equations for atherosclerotic cardiovascular disease risk [7].

Another area where ML and AI have shown promise is the early detection and prognosis of cancer. These techniques have been applied to predict cancer prognosis and estimate treatment response based on genomic and clinical data [8–14]. For instance, Lu et al. (2021) developed a deep learning network to predict early on-treatment response in metastatic colorectal cancer, which outperformed traditional methods [13].

ML and AI have also been employed to predict the onset and progression of neurodegenerative diseases, such as Alzheimer’s disease. By integrating neuroimaging, genetic, and clinical data, researchers have developed models that can identify individuals at high risk of developing Alzheimer’s disease [15–18]. Bhagwat et al. (2018) used a neural-network algorithm to predict the conversion from mild cognitive impairment to Alzheimer’s disease with as high as 0.90 accuracy [18].

In the realm of infectious diseases, ML and AI have been applied to predict disease outbreaks and identify high-risk areas [19, 20]. Bengtsson et al. (2015) utilized mobile phone data and machine learning techniques to predict the spatial spread of cholera in Haiti following the 2010 earthquake [20]. Similarly, these techniques have been used to assess the health impacts of environmental exposures, such as air pollution, by estimating daily pollutant concentrations and providing high-resolution exposure assessments for epidemiological studies [21]. Moreover, Odlum and Yoon (2015) leveraged natural language processing (NLP) techniques on data extracted from the social media platform Twitter to develop a real-time model for Ebola outbreak surveillance during the early stage of the 2014 epidemic [22]. Their study showcased the potential of applying advanced computational methods to unconventional data sources for enhanced disease monitoring and early detection.

Furthermore, ML and AI have been employed to investigate the social determinants of health and identify populations at high risk of adverse health outcomes [23]. By analyzing EHRs and integrating data on social and environmental factors, researchers have developed models that predict an individual’s risk of experiencing health disparities or poor health outcomes [24].

There are cases where ML and AI models in epidemiology have been successfully implemented to assist resource allocation and decision-making in practice. During the COVID-19 pandemic when the healthcare systems were strained by increased healthcare demand, clinicians in emergency departments faced significant challenges in patient disposition decision based on the patient’s initial symptoms and limited information. In response, Hinson et al. (2022) developed a ML algorithm to predict near-term clinical deterioration in emergency patients with real-time EHR data [25]. This tool was rapidly integrated into clinical practice to support care decisions within the Johns Hopkins Health System, contributing to more consistent and reliable disposition decision and improved bed allocation during the pandemic [26]. To inform resource allocation and enhance precision medicine in cardiovascular diseases, Ye et al. (2018) developed and validated a ML model on EHRs of more than 1.5 million individuals to predict the risk of incidence essential hypertension within the next year [27]. Demonstrating excellent performance, this model has been deployed in Maine, the United States, to assist healthcare providers in identifying high-risk populations and promoting individualized treatment [27].

In the end, ML and AI can also contribute to causal inference by identifying potential causal pathways and controlling for confounding factors in observational studies. For example, Kang et al. (2021) utilized a deep learning-based causal inference framework to estimate the causal effect of air pollution on COVID-19 severity while adjusting for confounding factors such as socioeconomic status and weather conditions [28]. Similarly, Chu et al. (2020) proposed an adversarial deep treatment effect prediction (ADTEP) model to predict treatment effects using heterogeneous EHR data [29].

In summary, the application of ML and AI in epidemiology offers several key benefits, including:

1. Handling large, complex datasets: ML and AI can process vast amounts of high-dimensional data, making them valuable tools for extracting meaningful insights from diverse data sources.
2. Identifying complex patterns: These techniques can uncover intricate, non-linear relationships between exposures and health outcomes, which may not be easily identified using traditional statistical methods.
3. Integrating multiple and multimodal data types: ML and AI can incorporate data from various sources, such as EHRs, genomic data, and environmental exposures, as well as structured and unstructured text, audio, image, and video data, to provide a more comprehensive understanding of the factors influencing health outcomes.
4. Improving predictive accuracy: These approaches often achieve higher predictive accuracy than traditional methods, particularly when dealing with complex datasets, enabling the development of more precise risk prediction models.
5. Enhancing causal inference: While primarily used for prediction, ML and AI can also contribute to causal inference by identifying potential causal pathways and controlling for confounding factors in observational studies.

By leveraging these benefits, the application of ML and AI in epidemiology has the potential to advance our understanding of disease risk factors, improve early detection and prognosis, and, thereby, inform targeted interventions to promote population health.

## Opportunities for ML and AI in life-course epidemiology

In life-course epidemiology that considers long-term effects of biological, behavioral, and social exposures during gestation, childhood, adolescence, and adulthood, ML and AI offer numerous opportunities by enabling researchers to identify sensitive periods, model complex interactions, predict disease risk trajectories, and enhance causal inference methods.

### Identifying sensitive periods and critical windows for intervention

ML and AI can help identify sensitive periods and critical windows for intervention by analyzing longitudinal data on growth and development of exposure and health outcomes. Unsupervised learning techniques, such as clustering and latent class analysis, can uncover distinct subgroups of individuals with similar developmental trajectories, which may inform the timing of interventions [30, 31]. Additionally, ML and AI allow for integration of multiple data types, including EHRs, genomic data, and environmental exposures, and of multimodal data, such as kinds of information from individual’s various types of records, thus providing a comprehensive perspective of the determinants of health outcomes across the different stages of life course [32]. Furthermore, potential causal pathways and mechanisms underlying the associations between exposures during critical windows of early life and later health outcomes can be better established by applying causal discovery algorithms or Mendelian randomization techniques [33].

### Modeling complex interactions between biological, social, and environmental factors

ML and AI techniques, such as deep learning and agent-based modeling, can capture the complex, non-linear associations between multiple risk factors and health outcomes across the life course. These approaches can help researchers understand how individual-level exposures and experiences at different life stages interact to shape population-level patterns of health and disease. For example, deep learning algorithms can model the hierarchical and temporal relationships between genetic susceptibility, early life adversity, and adult lifestyle factors to predict the risk of developing chronic diseases. Agent-based models can simulate the spread of infectious diseases through a population, taking into account individual susceptibility, contact patterns, and environmental conditions [34].

### Predicting individual and population-level disease risk trajectories

ML and AI can be used to develop personalized risk prediction models that estimate an individual’s likelihood of developing a particular disease based on their unique combination of risk factors and exposures across the lifespan. By effectively combining data from various sources such as genomic data, EHRs, and lifestyle factors, these models can provide accurate prediction of disease risks at different stages at an individual’s lifespan. At the population level, ML and AI can identify high-risk subgroups and distinct disease trajectories associated with specific combinations of early life exposures, socioeconomic factors, and health behaviors [4, 9, 16, 30, 31]. This information can guide the development of targeted interventions and policies to prevent and manage chronic diseases.

### Enhancing causal inference methods in observational studies

ML and AI techniques can strengthen causal inference methods in life-course epidemiology by helping researchers adjust for confounding factors and estimate causal effects in observational studies. Propensity score methods, which estimate the probability of an individual receiving a particular treatment or exposure based on their observed characteristics, can be enhanced using ML algorithms to more accurately balance the distribution of potential confounders between exposed and unexposed groups [35]. Instrumental variable methods, which use factors associated with the exposure but not the outcome to estimate causal effects, can be improved by using ML to identify and validate potential instrumental variables [36]. Additionally, advanced ML techniques, such as causal forests, can directly estimate heterogeneous treatment effects and minimize bias in observational studies [37–39].

By harnessing the power of ML and AI in these key areas, life-course epidemiology can gain novel insights into the complex determinants of health and disease across the lifespan, ultimately informing the development of more effective, personalized interventions and public health strategies.

## Framework for harnessing ML and AI technologies to advance public health solutions

The five principles outlined by Elder and Shanahan offer a robust conceptual framework for comprehending the intricate and ever-changing aspects of health and disease throughout an individual’s life course [2]. These principles also serve as a foundation for harnessing the potential of ML and AI to identify previously unknown risk factors, predict disease progression, and guide the development of targeted interventions. By leveraging the five principles of life-course research, we discuss the applications of ML and AI in life-course epidemiology based on the framework proposed by Elder and Shanahan [2].

1. Lifespan development: This principle emphasizes that human development and aging are lifelong processes, highlighting the importance of examining the cumulative effects of exposures and experiences across the entire life course. ML and AI techniques, such as deep learning and longitudinal modeling, can analyze large, complex datasets spanning multiple life stages to identify patterns and trajectories of health and disease over time [30, 31].
2. Agency: This principle recognizes that individuals have the capacity to make choices and take actions that shape their health and well-being within the constraints of their social and environmental contexts. ML and AI techniques, such as decision trees and reinforcement learning, can model the complex interactions between individual agency and social and environmental factors to identify key intervention points for promoting health and preventing disease [40].
3. Time and place: This principle emphasizes that every individual life course is embedded within and influenced by its specific historical and geographic context. ML and AI techniques, such as spatial modeling and time series analysis, can analyze the effects of place and time on health outcomes and identify key contextual factors that may influence the effectiveness of public health interventions [39, 41]. Recurrent neural networks (RNNs) and long short-term memory (LSTM) networks have been extensively employed for temporal data analysis, enabling the capture of dependencies and patterns over time [42–44]. Particularly, in recent years, novel architectures like Transformers have emerged and gained prominence due to their capacity to handle long-range dependencies and facilitate parallel processing. Transformers, exemplified by the Bidirectional Encoder Representations from Transformers (BERT) model, have exhibited superior performance across a wide range of natural language processing tasks and have been successfully adapted for time series analysis [45, 46].
4. Timing: This principle recognizes that the same events and experiences can have different effects on health depending on when they occur in the life course. ML and AI techniques, such as survival analysis (e.g. Survival Forest) and Bayesian networks, can model the time-varying effects of exposures on health outcomes and identify optimal timing and targeting of interventions [37, 38].
5. Linked lives: This principle emphasizes that individuals are embedded within social networks and relationships that shape their exposures, behaviors, and outcomes. ML and AI techniques, such as social network analysis and agent-based modeling, can model the complex interactions between individuals and their social environments to identify key leverage points for interventions that promote health and well-being across communities [40, 47]. Moreover, large language models (LLMs) can be integrated into this principle to analyze social media data and patient-generated content, providing insights into the social and environmental factors influencing health outcomes across communities and networks [23, 48–52].

The power of ML and AI techniques also enable itself to integrate multiple principles of the life-course approach simultaneously, enabling researchers to develop more comprehensive models of health trajectories. For instance, causal inference methods enhanced by ML, such as causal forests, can simultaneously address multiple principles by estimating heterogeneous treatment effects across different life stages, social contexts, and individual characteristics [37–39].

## Challenges and ethical considerations

The integration of ML and AI in life-course epidemiology presents several challenges and ethical considerations that must be addressed to ensure the responsible and effective use of these technologies.

### Data quality, harmonization, and integration

One major challenge is ensuring the quality, harmonization, and integration of data across multiple cohorts and sources [53]. Some data sources used for training ML models, such as EHRs collected for administrative purposes, might not be gathered with the necessary frequency, granularity, or bandwidth that align with information needs of science and learning, and may therefore present challenges in generating accurate and reliable algorithms [54]. Models trained on small sample sizes or data of suboptimal quality involving missing values, inaccuracies, and inconsistencies can lead to unreliable predictions and biased outcomes. Unlike certain health specialties such as dermatology or ophthalmology, where ML and AI have been successfully adopted due to their reliance on visual evaluation and pattern recognition [11], the application of ML and AI in epidemiology presents unique challenges. Life-course studies often involve data collected using different methods, at different time points, and from diverse populations. Ensuring the comparability and interoperability of these data is crucial for developing robust and generalizable ML and AI models. This requires close collaboration between researchers, data managers, and IT professionals to establish common data standards and protocols. Furthermore, life-course studies often involve factors with complex interactions and dynamic nature, as well as social phenomena that are inherently difficult to quantify and model. When dealing with dynamic variables, it is essential to retrain and reevaluate the models to account for new trends and changes, requiring ongoing monitoring and updates to maintain accuracy and relevance.

### Interpretability and explainability

Another significant challenge is the interpretability and explainability of ML and AI models. As these algorithms become increasingly complex, it can be difficult to understand how they arrive at their predictions or decisions. This “black box” nature of some ML models raises concerns about their transparency and accountability, particularly in the context of public health interventions that can have far-reaching consequences [55, 56]. Researchers must strive to develop models that are not only accurate but also interpretable, allowing for clear communication of their underlying logic and limitations to policymakers, healthcare providers, and the public. A case study indicated that when dealing with complex social factors, integrating predictive ML models with explanatory models can enhance understanding and prediction of outcomes [57].

### Bias and generalizability

Bias and generalizability are critical issues in the application of ML and AI to life-course epidemiology. If the training data used to develop these models are not representative of the broader population or if they contain historical biases, the resulting algorithms may perpetuate or even amplify these biases [58]. Results generated from these algorithms can lead to unintended consequences, such as the exacerbation of health disparities or the misallocation of resources. For example, EHR data are often a complex product of biological, socio-economic conditions as well as prior practices of providers and health systems [54]. Researchers must be vigilant in identifying and mitigating potential sources of bias in different data sources to ensure that their models are equitable and generalizable to diverse populations.

### Integration with domain knowledge

While ML and AI can identify novel patterns and associations in data, they do not necessarily provide insights into the underlying biological or clinical mechanisms. Integrating ML and AI findings with existing domain knowledge and expert interpretation is essential for ensuring the validity and relevance of the results [59, 60]. This requires close collaboration between data scientists, epidemiologists, and clinical experts, as well as a willingness to iterate between data-driven and hypothesis-driven approaches.

### Privacy and ethical concerns

The use of sensitive data in life-course studies poses significant privacy concerns and ethical challenges [61, 62]. These studies often involve the collection and analysis of highly personal information, such as genetic data, medical records, and social media activity. Ensuring the security and confidentiality of these data is paramount, requiring robust data governance frameworks and strict adherence to ethical guidelines. Researchers must also grapple with the potential unintended consequences of their work, such as the stigmatization of certain groups or the misuse of predictive models for discriminatory purposes. Strategies such as adopting synthetic data for training models provide new opportunities to improve the diversity and robustness of ML and AI models by reducing patient privacy concerns and facilitating data sharing while maintaining the original distribution of data [63].

### Computational resources and expertise

Applying ML and AI techniques to large-scale epidemiological data can be computationally intensive and require specialized expertise in data science and programming. Access to high-performance computing resources and qualified personnel may be a barrier for some research groups, particularly in low- and middle-income settings. Building capacity and infrastructure for ML and AI in epidemiological research is an important challenge that requires ongoing investment and support.

### Potential overreliance on ML and AI

While ML and AI offer significant opportunities for advancing research, clinical practice, and policymaking, it is crucial to recognize that not all ML and AI models outperform traditional models in healthcare and public health areas [64, 65]. A salient illustration of this phenomenon is the “Fragile Families Challenge,” wherein diverse state-of-the-art ML models were employed to predict individual life outcomes, including psychological and socio-economic status [66]. The resultant performance exhibited only marginal improvement over simple benchmark models [66]. This limited prediction value highlights the potential limitations of ML models when applied to complex social phenomena, contrasting with their success in physical and biological contexts [66, 67]. Prior to the adoption of ML and AI technologies, particularly in applied domains, it appears requisite to conduct a critical evaluation of these techniques vis-à-vis traditional models. This is especially pertinent in scenarios where reliable or superior alternatives are extant [64]. While a consensus has yet to emerge, such evaluation seems essential to preclude unnecessary investment in these sophisticated models and to mitigate excessive consumption of finite computational resources.

Despite the demonstrated methodological efficacy of ML and AI models, it is imperative to acknowledge that their implementation may not necessarily yield net positive outcomes for patients. A systematic review of 65 randomized controlled trials evaluating AI-based prediction tools revealed that approximately 40% of the tools, which had previously exhibited satisfactory performance in observational model development or validation studies, failed to demonstrate statistically significant clinical benefits for patients when compared to standard clinical care protocols [68]. The dynamic nature of clinical practice, fluctuations in the prevalence of comorbidities, and various socio-environmental factors can precipitate shifts in the distribution of patient characteristics. Consequently, these changes necessitate the periodic retraining and re-evaluation of AI systems to maintain their relevance and efficacy [69, 70].

Addressing these challenges and ethical considerations will require ongoing collaboration and dialogue among researchers, policymakers, and community stakeholders. It will be essential to develop guidelines and best practices for the responsible use of ML and AI in life course epidemiology, ensuring that these technologies are applied in a manner that is transparent, accountable, and aligned with public values and priorities. By proactively addressing these issues, we can harness the power of ML and AI to advance our understanding of health and disease across the life course while safeguarding the rights and wellbeing of individuals and communities.

## Future directions and recommendations

To fully realize the potential of ML and AI in life-course epidemiology and advance public health solutions, we discuss the following recommendations for future research and practice:

### Foster interdisciplinary collaborations

Collaboration between epidemiologists, data scientists, and public health professionals is crucial for the successful integration of ML and AI in life-course research. These collaborations will enable the exchange of knowledge, skills, and expertise necessary to develop and apply cutting-edge ML and AI techniques to complex life-course data. Epidemiologists contribute a deep understanding of the biological, social, and environmental factors influencing health trajectories, while data scientists bring advanced computational and analytical skills. Public health professionals provide invaluable insights into the practical implications and translational potential of research findings. By working together, these multidisciplinary teams can drive innovation, uncover novel insights, and ultimately improve population health outcomes.

### Develop standardized guidelines and best practices

Establishing standardized guidelines and best practices for using ML and AI in life-course research is essential to ensure the reliability, reproducibility, and ethical application of these technologies. Clear protocols should be developed for data collection, preprocessing, and analysis, as well as guidelines for model development, validation, and reporting. These standards should be created through a collaborative process involving researchers, professional societies, and other stakeholders, drawing on existing best practices in epidemiology, data science, and bioethics. By promoting transparency, consistency, and rigor in the use of ML and AI, the credibility and impact of life course research can be enhanced. In the meantime, it is important to be aware of the limits of ML and AI applications. Guidelines should be formulated to instruct the necessity and appropriateness of adopting ML and AI in different areas.

### Advocate for the integration of ML and AI in public health decision-making

Translating research findings into actionable policies and interventions is the key to realizing the full potential of ML and AI in life-course epidemiology. This requires close collaboration between researchers, policymakers, and community stakeholders to ensure that ML and AI models are developed and applied in a manner that addresses real-world health challenges and promotes health equity [71]. In order to generate results that can translate to advances that promote population health and health systems, the learning initiatives must align with questions that impact the important aspects of clinical practice or health-related decision-making [54]. Researchers should strive to communicate their findings in a clear, accessible language and engage with decision-makers and the public to build trust and understanding of these technologies. Policymakers, in turn, should invest in the infrastructure and resources necessary to support the use of ML and AI in public health, including data systems, computational tools, and workforce development. By working together, the power of ML and AI can be harnessed to inform evidence-based policies and interventions that improve health outcomes across the life course.

### Prioritize equity and fairness in ML and AI applications

As ML and AI technologies become increasingly integrated into life-course research and public health practice, it is crucial to prioritize equity and fairness in their development and application [58, 72]. Researchers should assess the data comprehensively given the specific purposes, while actively work to identify and mitigate potential sources of bias in their data and models, ensuring that the benefits of these technologies are distributed equitably across diverse populations. This may involve developing new methods for bias detection and correction, as well as engaging with communities and stakeholders to understand their needs and concerns. Policymakers and funding agencies should also prioritize research and initiatives that focus on addressing health disparities and promoting health equity through the use of ML and AI.

### Invest in training and capacity building

To fully capitalize on the potential of ML and AI in life-course epidemiology, it is essential to invest in training and capacity building for researchers, public health professionals, and policymakers. This may involve developing new educational programs and curricula that integrate data science and computational skills with domain expertise in epidemiology and public health. It may also require establishing new funding mechanisms and support structures to enable researchers to access the computational resources and expertise necessary to apply ML and AI techniques to their data. Building a skilled and diverse workforce that can effectively leverage these technologies will be critical for driving innovation and progress in life course research and public health practice.

By pursuing these recommendations and prioritizing interdisciplinary collaboration, standardization, integration, equity, and capacity building, the field of life course epidemiology can harness the full potential of ML and AI to advance our understanding of health and disease across the lifespan and develop more effective, equitable, and evidence-based public health solutions.

## Conclusions

The integration of ML and AI in life-course epidemiology presents a remarkable opportunity to advance our understanding of the complex interplay between biological, social, and environmental factors that shape health trajectories across the lifespan. Leveraging these powerful technologies to analyze diverse datasets can yield novel insights, improve disease risk prediction, and inform the development of targeted interventions.

However, the realization of this potential is contingent upon addressing the significant challenges associated with the use of ML and AI, including issues related to data quality, model interpretability, bias, privacy, and equity. To fully harness the power of these technologies, it is imperative to foster interdisciplinary collaborations, establish standardized guidelines and best practices, advocate for the integration of ML and AI into public health decision-making processes, prioritize fairness in their application, and invest in training and capacity building.

It is important to acknowledge that this perspective paper, while striving for a balanced and comprehensive discussion, is not a systematic review. As such, the information presented may be subject to selection bias. Our narrative approach, while broad, may not capture all relevant studies or viewpoints. The examples cited were selected based on relevance and impact, but may not represent an exhaustive body of evidence. Readers should consider this limitation when interpreting the conclusions and recommendations presented.

As we look to the future, we should be guided by a vision of harnessing data, technology, and innovation to promote health, prevent disease, and reduce inequities across the life course. By working collaboratively to responsibly integrate ML and AI into life-course epidemiology, we can take a significant step towards creating a healthier and more equitable future for all.

## Data Availability

Not applicable.

## Abbreviations

AI: Artificial intelligence
EHR: Electronic health record
LLM: Large language models
ML: Machine learning
NLP: Natural language processing

## Data availability

No datasets were generated or analysed during the current study.
